# S-1-Based Chemotherapy versus Capecitabine-Based Chemotherapy as First-Line Treatment for Advanced Gastric Carcinoma: A Meta-Analysis

**DOI:** 10.1371/journal.pone.0082798

**Published:** 2013-12-12

**Authors:** Ming-ming He, Wen-jing Wu, Feng Wang, Zhi-qiang Wang, Dong-sheng Zhang, Hui-yan Luo, Miao-zhen Qiu, Feng-hua Wang, Chao Ren, Zhao-lei Zeng, Rui-hua Xu

**Affiliations:** Department of Medical Oncology and State Key Laboratory of Oncology in South China, Sun Yat-sen University Cancer Center, Guangzhou, Guangdong, China; Sudbury Regional Hospital, Canada

## Abstract

**Background:**

Although both oral fluoropyrimidines were reported effective and safe, doubts exist about whether S-1 or capecitabine is more advantageous in advanced gastric carcinoma (AGC). Herein, we performed a meta-analysis to comprehensively compare the efficacy and safety of S-1-based chemotherapy versus capecitabine-based chemotherapy as first-line treatment for AGC.

**Methods:**

PubMed/Medline, EmBase, Cochrane library, and China National Knowledge Infrastructure databases were searched for articles comparing S-1-based chemotherapy to capecitabine-based chemotherapy for AGC. Primary outcomes were overall response rate (ORR), time to progression (TTP), overall survival (OS), progression-free probability, and survival probability. Secondary outcomes were toxicities. Fixed-effects model were used and all the results were confirmed by random-effects model.

**Results:**

Five randomized controlled trials and five cohort studies with 821 patients were included. We found equivalent ORR (38.3% vs. 39.1%, odds ratio [OR] 0.92, 95% conﬁdence interval [CI] 0.69-1.24, *P* = 0.59), TTP (harzad ratio [HR] 0.98, 95% CI 0.82-1.16, *P* = 0.79), OS (HR 0.99, 95% CI 0.87-1.13, *P* = 0.91), progression-free probability (3-month OR 1.02, 95% CI 0.62-1.68, *P* = 0.94; 6-month OR 1.34, 95% CI 0.88-2.04, *P* = 0.18) and survival probability (0.5-year OR 0.90, 95% CI 0.61-1.31, *P* =0.57; 1-year OR 0.97, 95% CI 0.70- 1.33, *P* = 0.84; 2-year OR 1.15, 95% CI 0.61-2.17, *P* = 0.66). Equivalent grade 3 to 4 hematological and non-hematological toxicities were found except hand-foot syndrome was less prominent in S-1-based chemotherapy (0.3% vs. 5.9%, OR 0.19, 95% CI 0.06-0.56, *P* = 0.003). There’re no significant heterogeneity and publication bias. Cumulative analysis found stable time-dependent trend. Consistent results stratified by study design, age, regimen, cycle, country were observed.

**Conclusion:**

S-1-based chemotherapy was associated with non-inferior antitumor efficacy and better safety profile, compared with capecitabine-based therapy. We recommended S-1 and capecitabine can be used interchangeably for AGC, at least in Asia.

## Introduction

Gastric carcinoma ranks second among the most common causes of cancer deaths worldwide, with especial high prevalence in Asia [1-3]. A large number of gastric cancer patients present with advanced disease (unresectable, recurrent or metastatic disease) precluding surgery and chemotherapy becomes the most effective treatment [4-6]. However, a globally accepted standard regimen has not been established, among which fluoropyrimidines comprise the backbone of chemotherapy for advanced gastric carcinoma (AGC) and the optimization was established by extensive research [7,8]. Oral fluoropyrimidines (capecitabine and S-1) have opened new perspectives for treatment for AGC with their simplicity and convenience over the traditional 5-FU [9-11].

Capecitabine was suggested as a suitable alternative for 5-FU in AGC in REAL 2 trial [12], ML17032 trial [13], and two meta-analyses with a superior overall survival (OS) versus 5-FU in AGC (harzad ratio (HR) 0.87, 95% conﬁdence interval (CI) 0.77-0.98) [14] and in gastrointestinal cancers (HR 0.94, 95% CI 0.89-1.00) [15]. By now, capecitabine-based combinations have become the standard treatment for AGC globally. 

S-1 is another preferred oral ﬂuoropyrimidine for AGC. Randomized trials, comparing S-1 with 5-FU in mono (JCOG 9912 [16]) or combined therapy (FLAGS trial [17] and SC-101 study [18]), have revealed a non-inferior efficacy and better toxicity profile. A meta-analysis showed OS favored S-1-based chemotherapy over 5-FU-based chemotherapy in AGC (HR 0.87, 95% CI 0.79-0.96) [19]. S-1-based combinations are widely used for AGC in Asia and recently in European countries.

However, doubts exist about whether S-1 or capecitabine is more advantageous in first-line treatment for AGC. Several clinical trials and cohort studies, comparing S-1 with capecitabine in mono or combined therapy, have published no completely consistent results. Some slightly favored S-1 on efficacy [20], some slightly favored capecitabine [21,22], while some reported equivalent results [23,24]. No consensus on toxicity profiles of these two chemotherapies were reached especially on hand-foot syndrome, thrombocytopenia, stomatitis and diarrhea [20,22-25]. These allowed no definite conclusions about the efficacy and safety of these two chemotherapies with limited number of individuals assessed. In addition, the non-uniform study design, regimen, chemotherapy cycle, patient age and country all made people assailed with doubts. Meanwhile, there has been no meta-analysis to detect the difference of these two oral fluoropyrimidines in any cancer.

Evaluation of the efficacy and safety of these two oral fluoropyrimidines will provide necessary and important information for making clinical decision. Therefore, we conducted a meta-analysis with greater power of statistical comparisons to comprehensively compare S-1-based chemotherapy versus capecitabine-based chemotherapy as first-line chemotherapy for AGC.

## Materials and Methods

### Search Strategy

To ensure retrieval of all relevant studies, two authors (Ming-ming He and Wen-jing Wu) used a broad search strategy independently with text words “gastric/stomach/gastrointestinal/ gastroesophageal/esophagogastric/intestinogastric/gastroenterological,” “cancer/carcinoma/tumor/neoplasm/adenocarcimoma,” “S-1/TS-1/Tegafur Gimeracil Oteracil/oteracil/gimeracil/,” and “capecitabine/xeloda” in PubMed/Medline, EmBase, Cochrane Library and China National Knowledge Infrastructure databases [2,26]. An additional search through Google Scholar and manual search through published literatures were used for supplementation. The references of the identified articles were checked. Corresponding authors were contacted for further details if necessary. Discrepancies were resolved by the third party (Rui-hua Xu, Feng Wang) adjudication. To limit publication bias, no language limitation, time limitation or other restrictions such as study design were imposed [27].

### Selection Criteria

The inclusion criteria were as follows: (1) studies aimed to compare efficacy or safety between S-1-based chemotherapy and capecitabine-based chemotherapy as first-line chemotherapy for patients with advanced gastric adenocarcinoma (unresectable, recurrent or metastatic gastric cancer); (2) data for calculating the efficacy or safety of these two therapies were provided; (3) randomized controlled trials (RCTs), cohort studies. The exclusion criteria were: (1) studies with no data for efficacy and safety including protocols and phase 1 clinical trials; (2) studies based on overlapping patients; (3) Case reports, abstracts, reviews , conference reports and experiments.

### Data Extraction and Outcomes

We extracted data for demography information and potential confounding factors. Primary outcomes were overall response rate (ORR), time to progression (TTP), overall survival (OS), progression-free probability, survival probability. Secondary outcomes were toxicities. Figures were electronically digitized and Kaplan-Meier curves were downloaded by an appropriate software GetData Graph Digitizer (http://getdata-graph-digitizer.com). The data collection was in accordance with the Quality of Reporting of Meta-Analyses statement. We used the modified Jadad Scale [28] and the modified Newcastle-Ottawa scale [29] to assess the quality of RCTs and cohort studies, respectively.

### Statistical Analysis

All analyses were performed using the STATA 11.0 package (StataCorp, College Station, TX, USA). HR with 95% CI was used for TTP and OS as demonstrated by Parmar MK et al [30]. For binary data, including ORR, progression-free probability, survival probability and toxicities, the odds ratio (OR) with 95% CI was used. HR > 1 reﬂects more deaths or progression in the S-1-based arm. OR > 1 reﬂects a favorable outcome in the S-1-based arm for response, survival probability or an unfavorable outcome for toxicities. Fixed-effects model was used and we then used random-effects model to confirm all the results. Cumulative meta-analysis is performed to sort out the time-tendency of outcomes and meta-regression is performed to explain some heterogeneity. Subgroup analyses were conducted by potential confounding factors selected by reviewing the characteristics of included studies. Sensitivity analyses were conducted to assess the stability of the result. *P* < 0.05 was considered signiﬁcant. Heterogeneity was assessed by *I* ^*2*^ inconsistency test and χ^2^-based Cochran’s Q statistic test in which *I* ^2^ >50%, or *P* < 0.05 indicated signiﬁciant heterogeneity. Publication bias was detected by graphical funnel plots. Asymmetry of the funnel plot was tested by Begg’s test and Egger's test [31,32]. *P* < 0.05 was considered as signiﬁcant. This article follows the QUORUM and the Cochrane Collaboration guidelines (http://www.cochrane.de) for reporting meta-analysis and accords with the preferred reporting items for systematic reviews and meta-analyses (PRISMA) guidelines (Checklist S1).

## Results

### Eligible Studies

**Figure 1:**
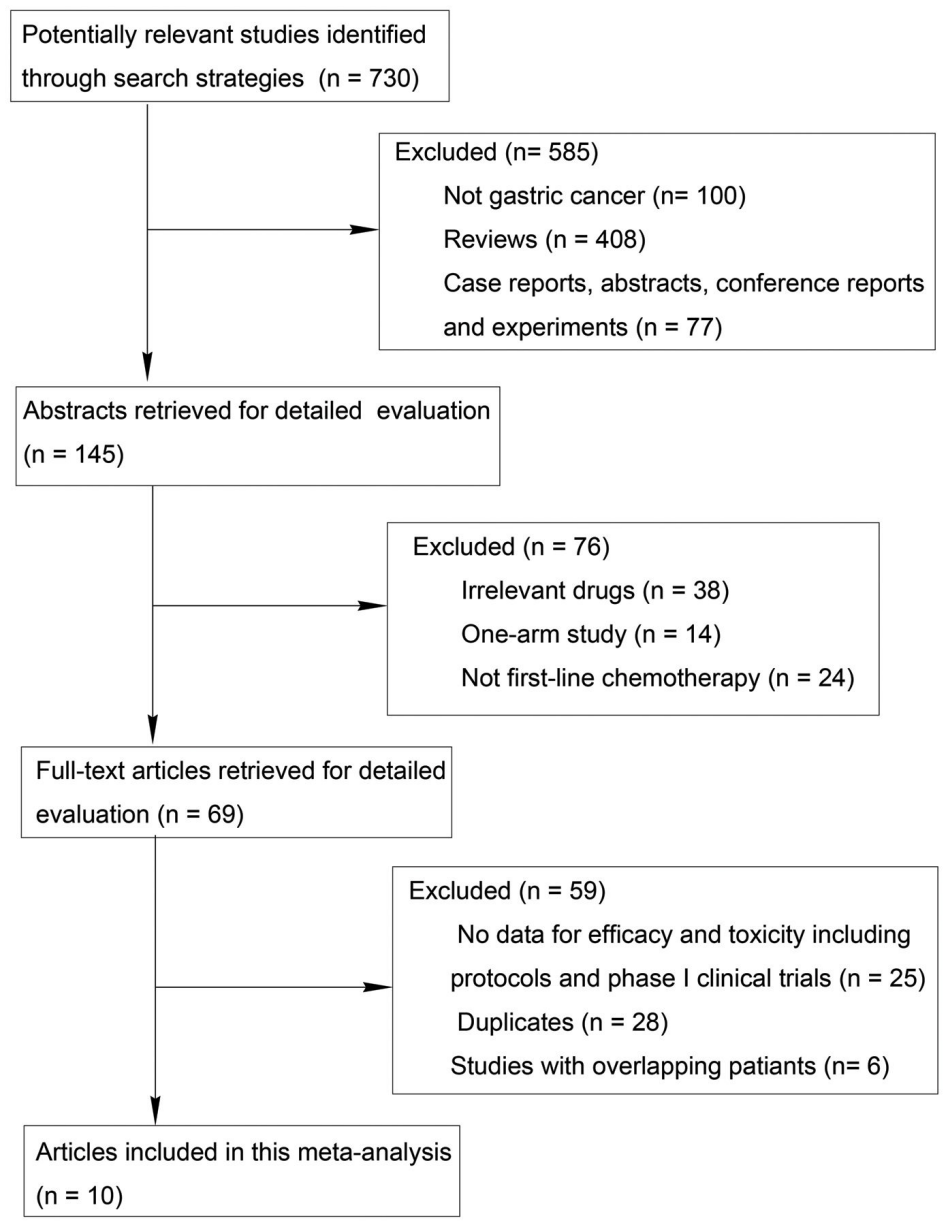
Meta-analysis profile summarizing trial flow.

Detailed steps of the search are shown (Figure 1). After the selection procedure, five RCTs [22,23,33-35] and five cohort studies [20,21,24,25,36] were included, with a total of 423 patients in S-1-based arm and 398 patients in capecitabine-based arm (Table 1). There were no significant differences in the baselines between S-1-based arm and capecitabine-based arm in these studies, as reported. All the RCTs were considered to be of high quality (Table 2) and the included cohorts showed satisfactory quality with reasonable selection criteria, comparable patient characteristics and adequate follow-up of the subjects (Table 3). 

**Table 1:** Basic characteristics of the studies included in this meta-analysis.

Study	Period	Country	Study design	Regimen	n	Age	Median cycles
Kim GM 2012	2008-2009	Korea	RCT	**S-1** 40 mg/m^2^ bid days 1-14, LOHP130 mg/m^2^ day1, q3w	65	60	6
				Capecitabine 1000mg/m^2^ bid days 1-14, LOHP 130 mg/m^2^ day1, q3w	64	61	8
Lee JL 2008	2004-2006	Korea	RCT	**S-1**40,50,or 60mg/m^2^ bid days 1-28 q6w	45	71	2
				Capecitabine 1250 mg/m^2^ bid days 1-14 q3w	46	71	5
Lim do H 2010	2008-2008	Korea	cohort	**S-1** 40 mg/m^2^ bid days 1-21, DDP 60-100 mg/m^2^ day1, q5w	97	53	5
				Capecitabine 1000 mg/m^2^ bid days 1-14, DDP 60-100 mg/m^2^ day1, q3w	77	59	4
Seol YM 2009	2004-2008	Korea	cohort	**S-1** 50 or 60 mg/m^2^ bid days 1-14, DDP 70 mg/m^2^ day1, q3w	32	73	6
				Capecitabine 1250 mg/m^2^ bid days 1-14, DDP 70 mg/m^2^ day1, q3w	40	74	6
Shitara K 2012	2006-2008	Japan	cohort	**S-1** 80 mg/m^2^ days 1-21, DDP 60 mg/m^2^ day1, 5w	50	61	4
				Capecitabine 1000 mg/m^2^, bid days 1-21, DDP 80 mg/m^2^ day1, q3w	26	65	6
Ba N 2012	2009-2010	China	RCT	**S-1** 40 mg/m^2^ bid days 1-14, DDP 75 mg/m^2^ day1, q3w	18	54	6
				Capecitabine 1000 mg/m^2^ bid days 1-14,DDP 75 mg/m^2^ day1, q3w	19	53	6
Gao W 2012	2008-2011	China	cohort	**S-1**40,50,or 60mg/m^2^ bid days 1-28 q6w	30	72.9	unknown
				Capecitabine 1250 mg/m^2^ bid days 1-14 q3w	26	73.5	unknown
Lu HF 2012	2009-2011	China	cohort	**S-1** 40 mg/m^2^ bid days 1-14, LOHP130 mg/m^2^ day1, q3w	31	68	5 (mean)
				Capecitabine 200 mg/m^2^ bid days 1-14, LOHP 130 mg/m^2^ day1, q3w	41	67	5 (mean)
Xiong HL 2013	2010-2011	China	RCT	**S-1** 40 mg/m^2^ bid days 1-14, Docetaxel 25 mg/m^2^day1,8,15, q4w	42	<65	3 for all
				Capecitabine 1250 mg/m^2^ bid days 1-14, Docetaxel 25 mg/m^2^day1,8,15, q4w	44	<65	3 for all
Yan SN 2012	2010-2011	China	RCT	**S-1**40,50,or 60mg/m^2^ bid days 1-14 q3w	15	73	3
				Capecitabine 1250 mg/m^2^ bid days 1-14, q3w	15	73	3

Abbreviations: **LOHP**, oxaliplatin; **DDP**, cisplatin; **RCT**, randomized controlled trial.

**Table 2:** Quality assessment of RCTs by modified Jadad scale†.

Study	Design	Randomiza-tion	Allocation concealment	Blinding	Loss to follow up	Number of dropout	Score	Quality
Ba N 2012	RCT	2	1	1	1	1	6	high
Kim GM 2012	RCT	2	2	1	1	1	7	high
Lee JL 2008	RCT	2	2	1	1	1	7	high
Xiong HL 2013	RCT	2	1	1	1	1	6	high
Yan SN 2012	RCT	2	1	1	1	1	6	high

^†^ There are four items in the Jadad scale: randomizations, allocation concealment, double blinding, withdrawals and dropouts. If the item was not described in the study, the score would be 0; otherwise it was 1. And if the method of the item was described and it was appropriate, the score would reach to 2 except for the item of withdrawals and dropouts. Randomized control trials (RCTs) were considered to be of high quality if the score was 4-7, of low quality if the score was 1-3.

**Table 3:** Modified Newcastle Ottawa quality assessment scale for cohort studies†.

Study	Selection				Comparability	Outcome		
	Representa-tiveness of the exposed cohort	Selection of the non-exposed cohort	Ascertain-ment of exposure	Incident disease		Assessm-ent of outcome	Length of follow-up	Adequacy of follow-up
Seol YM 2009	A	A	A	A	A	B	A	A
Lim Do H 2010	A	A	A	A	A	B	A	A
Shitara K 2012	A	B	A	A	A	B	A	A
Lu HF 2012	A	A	A	A	A	B	A	A
Gao W 2012	A	A	A	A	A	B	A	A

^†^ The Newcastle Ottawa scale is for case-control study and cohort study.

Selection: (1) Representativeness of the exposed cohort: A, truly representative of the average patient with S-1 regimen; B, somewhat representative of the average patient with S-1 regimen; C, selected group; and D, no description of the derivation of the cohort (2). Selection of the non-exposed cohort: A, drawn from the same community as the exposed cohort; B, drawn from a different source; and C, no description of the derivation of the non-exposed cohort (3). Ascertainment of exposure: A, secure record; B, structured interview; C, written self-report; and D, no description (4). Demonstration that outcome of interest was not present at the start of the study: A, yes; B, no; C, no description.

Comparability: Comparability of cohorts on the basis of the design or analysis: A, study controls for co-morbidities; B, study controls for additional risk factors (such as age, or severity of illness, etc,); C, not done.

Outcome: (1) Assessment of outcome: A, independent blind assessment; B, record linkage; C, self-report; D, no description (2). Was follow-up long enough for outcomes to occur: A, yes; B, no (3). Adequacy of follow-up of cohorts: A, complete follow-up—all subjects accounted for; B, subjects lost to follow-up unlikely to introduce bias (small number lost), follow-up rate higher than 90%, or description provided for those lost; C, follow-up rate 90% or lower and no description of those lost; D, no statement.

### Overall Response Rate

All the ten studies demonstrated ORR. The ORR of the S-1-based arm ranged from 20.0% to 50.0%, while the ORR of capecitabine-based arm ranged from 13.3% to 55.0%. The meta-analysis showed an equivalent ORR between S-1-based chemotherapy and capecitabine-based chemotherapy (38.3% vs. 39.1%, OR 0.92, 95% CI 0.69-1.24, *P* = 0.59; *I* ^2^ = 0%). 

**Figure 2:**
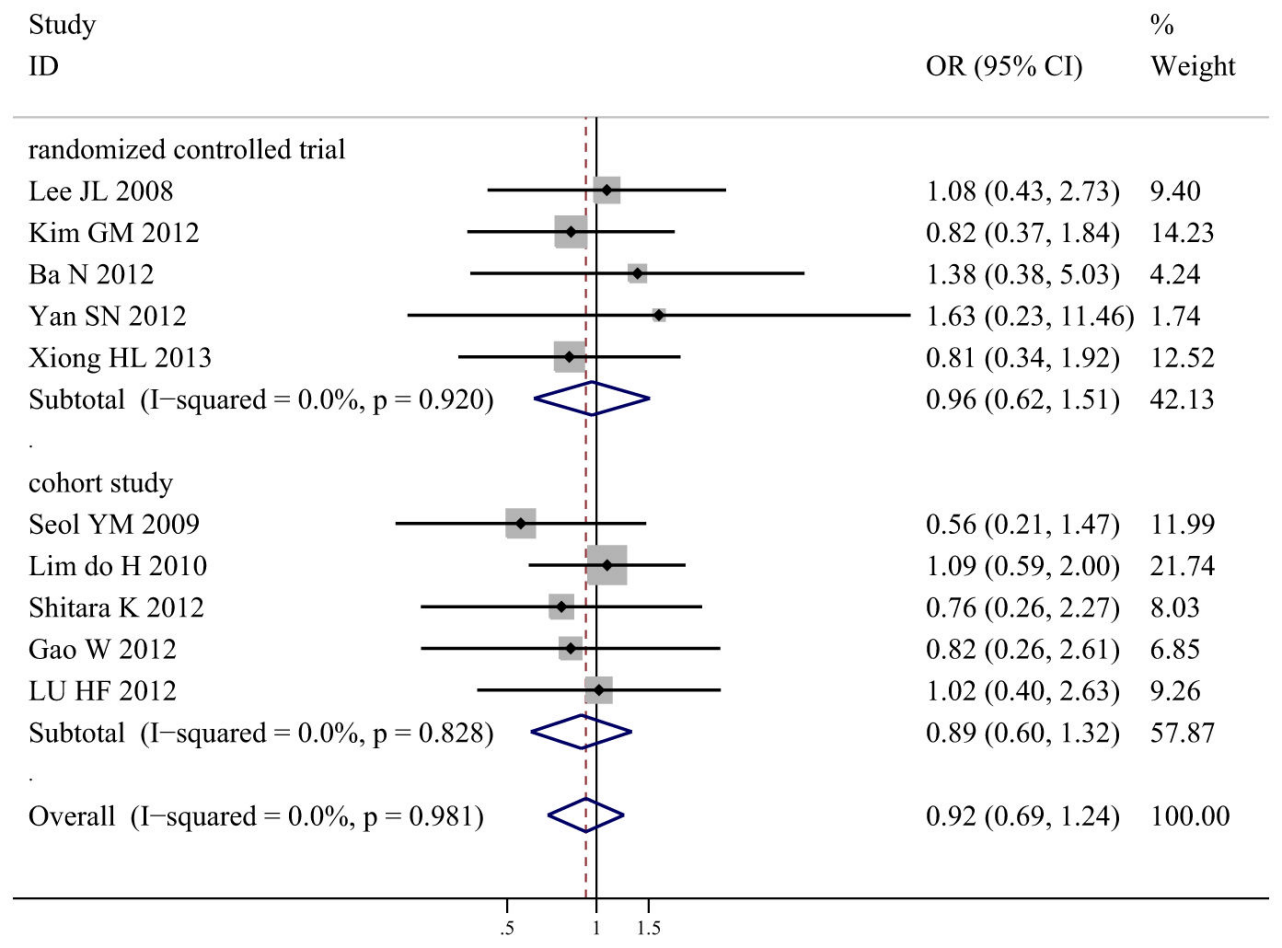
Meta-analysis of overall response rate for S-1-based chemotherapy compared with capecitabine-based chemotherapy.

Subgroup analysis according to study design found consistent result in the RCTs (OR 0.97, 95% CI 0.62-1.51, *P* = 0.88; *I* ^2^ = 0%) with the overall effect. Similarly, consistent result was also found in the cohort studies (OR 0.89, 95% CI 0.60-1.32, *P* = 0.57; *I* ^2^ = 0%) with the overall effect. The details were showed in Figure 2.

### Time to Progression

**Figure 3:**
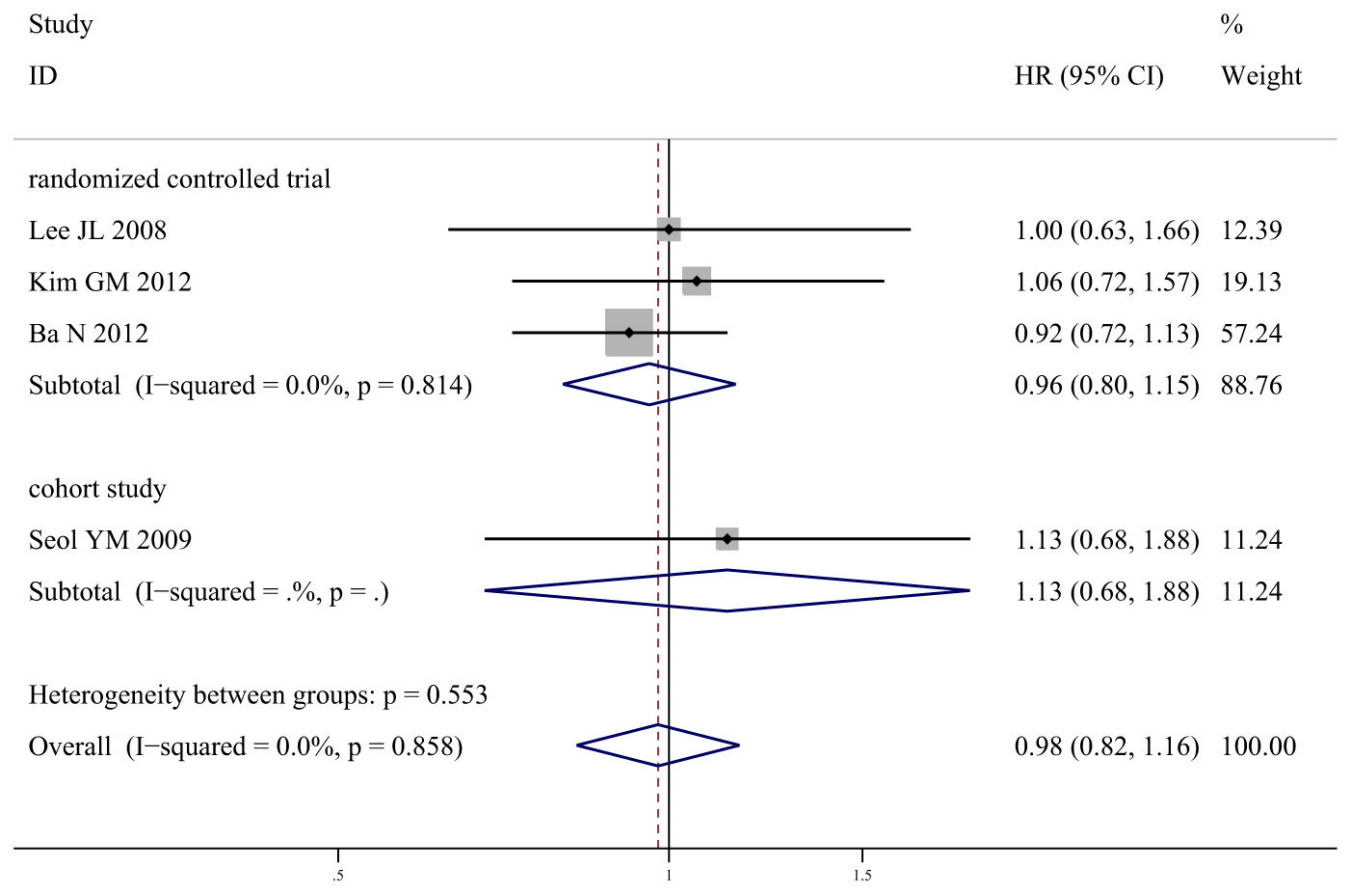
Meta-analysis of time to progression for S-1-based chemotherapy compared with capecitabine-based chemotherapy.

Five studies demonstrated median TTP which ranged from 4.2 to 6.2 months for S-1-based arm, and from 4.3 to 7.2 months for capecitabine-based arm. The pooled HR for TTP from 4 studies showed no signiﬁcant difference between S-1-based chemotherapy and capecitabine-based chemotherapy (HR 0.98, 95% CI 0.82-1.16, *P* = 0.79; *I* ^2^ = 0%). Similarly, no significant difference was found between the two arms in the subgroups of 3 RCTs (HR 0.96, 95% CI 0.80-1.15, *P* = 0.66; *I* ^2^ = 0%) and 1 cohort study (HR 1.13, 95% CI 0.68-1.88, *P* = 0.64) (Figure 3).

Another study demonstrated HR for progression-free survival (PFS) (median PFS 5.8 vs. 5.2 months, HR 0.97, 95% CI 0.60-1.58). A meta-analysis of pooled HR of the TTP and PFS together (TTP_PFS) still showed no signiﬁcant difference between the two arms (HR 0.98, 95% CI 0.83–1.15; *I* ^2^ = 0%) (Figure S1). 

### Progression-Free Probability

**Figure 4:**
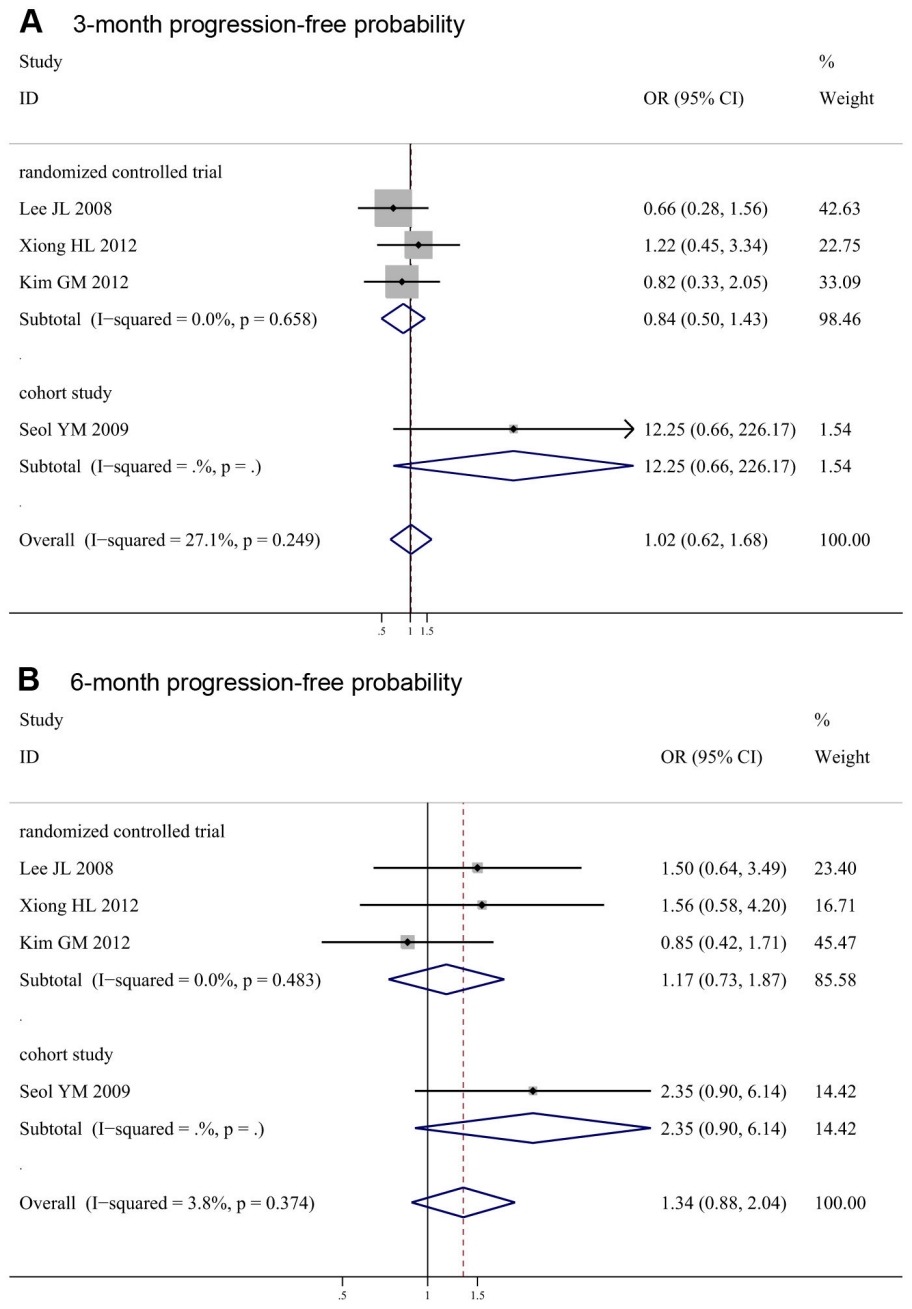
Meta-analysis of 3-month, 6-month progression-free probability for S-1-based chemotherapy compared with capecitabine-based chemotherapy.

The above four studies published Kaplan-Meier curves of time-to-progression. The meta-analysis indicated there were no significant differences between the two arms in 3-month progression-free probability (OR 1.02, 95% CI 0.62-1.68, *P* = 0.94; *I* ^2^ = 27%), and 6-month progression-free probability (OR 1.34, 95% CI 0.88-2.04, *P* = 0.18; *I* ^2^ = 4%) (Figure 4). 

In detail, meta-analysis of 3 RCTs also showed no significant differences between two arms (3-month OR 0.84, 95% CI 0.50-1.43, *P* = 0.53; *I* ^2^ = 0%; 6-month OR 1.17, 95% CI 0.73-1.87, *P* = 0.52; *I* ^2^ = 0%). Only 1 cohort study documented progression-free probability and also found no significant differences (3-month OR 12.25, 95% CI 0.66-226.17, *P* = 0.09; 6-month OR 2.35, 95% CI 0.90-6.14, *P* = 0.08).

### Overall Survival

**Figure 5:**
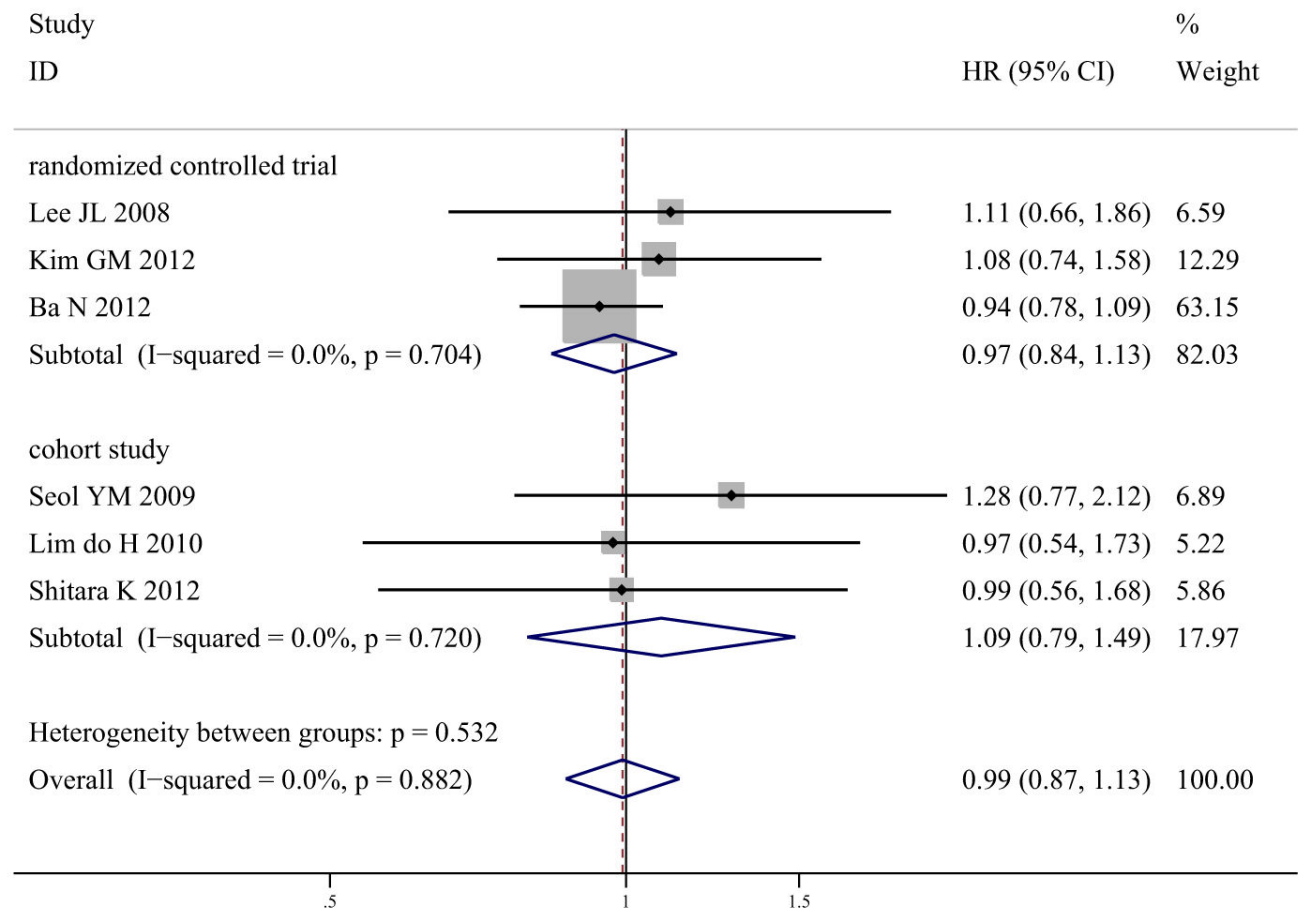
Meta-analysis of overall survival for S-1-based chemotherapy compared with capecitabine-based chemotherapy.

Median OS was demonstrated in eight studies which ranged from 7.8 to 13.8 months for S-1-based arm, and from 8.1 to 13.5 months for capecitabine-based arm. The pooled HR for OS of 6 studies showed no signiﬁcant difference between the two arms (HR 0.99, 95% CI 0.87–1.13, *P* = 0.79; *I* ^2^ = 0%) (Figure 5).

In detail, meta-analysis of 3 RCTs showed no significant difference of OS between two arms (HR 0.97, 95% CI 0.84–1.13, *P* = 0.71; *I* ^2^ = 0%). Meta-analysis of 3 cohort studies also showed no significant difference (HR 1.09, 95% CI 0.79–1.49, *P* = 0.61; *I* ^2^ = 0%).

### Survival Probability

**Figure 6:**
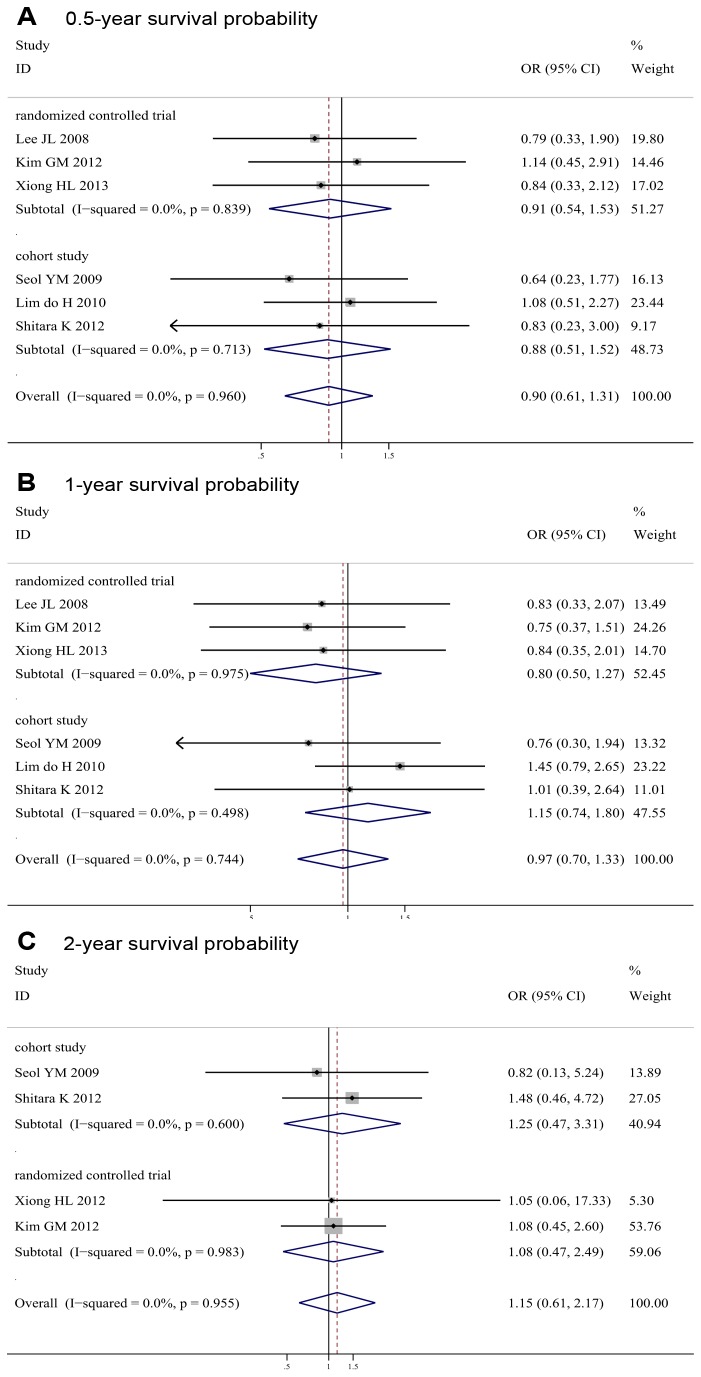
Meta-analysis of 0.5-year, 1-year, and 2-year survival probability for S-1-based chemotherapy compared with capecitabine-based chemotherapy.

The above six studies published Kaplan-Meier curves of overall survival. Meta-analysis of 0.5-, 1-, and 2-year survival probability found no significant differences between the two arms (0.5-year OR 0.90, 95% CI 0.61-1.31, *P* = 0.57; *I* ^2^ = 0%; 1-year OR 0.97, 95% CI 0.70-1.33, *P* = 0.84; *I* ^2^ = 0%; 2-year OR 1.15, 95% CI 0.61-2.17, *P* = 0.66; *I* ^2^ = 0%) (Figure 6).

Consistently, no significant differences of survival probability between the two arms were found in RCTs (0.5-year OR 0.91, 95% CI 0.54-1.53, *P* = 0.71; *I* ^2^ = 0%; 1-year OR 0.80, 95% CI 0.50-1.27, *P* = 0.34; *I* ^2^ = 0%; 2-year OR 1.08, 95% CI 0.47-2.49, *P* = 0.86; *I* ^2^ = 0%), and in cohort studies (0.5-year OR 0.88, 95% CI 0.51-1.52, *P* = 0.66; *I* ^2^ = 0%; 1-year OR 1.15, 95% CI 0.74-1.80, *P* = 0.53; *I* ^2^ = 0%; 2-year OR 1.25, 95% CI 0.47-3.31, *P* = 0.65; *I* ^2^ = 0%).

### Grade 3 to 4 Toxicities

Meta-analysis of grade 3 to 4 hematological and non-hematological toxicities found no significant differences between the two arms except hand-foot syndrome was less prominent for S-1-based chemotherapy (0.3% vs. 5.9%, OR 0.19, 95% CI 0.06-0.56, *P* = 0.003; *I* ^2^ = 0%) (Table 4). Similar results were found in meta-analysis of 4 RCTs (0.6% vs. 7%, OR 0.18, 95% CI 0.04-0.69, *P* = 0.01; *I* ^2^ = 0%). However, meta-analysis of 4 cohort studies showed the difference was not significant (0% vs. 4.5%, OR 0.21, 95% CI 0.04-1.24, *P* = 0.09; *I* ^2^ = 0%).

**Table 4:** Outcomes of toxicity meta-analysis compairing S-1-based chemotherapy versus capecitabine-based chemotherapy as first-line treatment in advanced gastric carcinoma.

Toxicity	Studies	Heterogeneity *P* value	Heterogeneity *I*2	OR (95%CI)	*P* value
Grade 3–4 leukopenia	5	0.97	0%	1.73 (0.73-4.13)	0.22
Grade 3–4 netropenia	7	0.84	0%	0.76 (0.45-1.27)	0.29
Grade 3–4 anemia	8	0.70	0%	1.23 (0.72-2.10)	0.44
Grade 3–4 thrombocytopenia	6	0.99	0%	1.02 (0.49-2.14)	0.95
Grade 3–4 febrile neutropenia	3	0.66	0%	1.06 (0.22-5.15)	0.94
Grade 3–4 asthenia	6	0.71	0%	0.72 (0.34-1.51)	0.38
Grade 3–4 anorexia	5	0.89	0%	1.26 (0.60-2.64)	0.53
Grade 3–4 nausea	7	0.72	0%	0.96 (0.50-1.86)	0.91
Grade 3–4 vomiting	4	0.87	0%	1.19 (0.34-4.22)	0.79
Grade 3–4 abdominal pain	2	0.57	0%	2.87 (0.71-11.64)	0.14
Grade 3–4 stomatitis	3	0.70	0%	1.12 (0.23-5.54)	0.89
Grade 3–4 diarrhea	8	0.68	0%	0.84 (0.38-1.90)	0.68
Grade 3–4 hand–foot syndrome	8	0.99	0%	0.19 (0.06-0.56)	0.003
Grade 3–4 neuropathy	3	0.67	0%	0.80 (0.17-3.64)	0.77
Grade 3–4 infection	2	0.90	0%	1.47 (0.45-4.85)	0.52

Abbreviations: **OR**, odds ratio; **CI**, conﬁdence interval.

### Heterogeneity, Regression Analysis and Publication Bias Assessment

No significant heterogeneity was found for all analyses (*I* ^2^ < 50%, *P* > 0.05), When fixed-effects model changed to random-effects model for all comparisons, all the results remain.

Meta-regression analysis further found patient median age of either group was not significant contributor to between-study heterogeneity (*P* ranging from 0.283 to 0.876), without enough information for other string variables.

**Figure 7:**
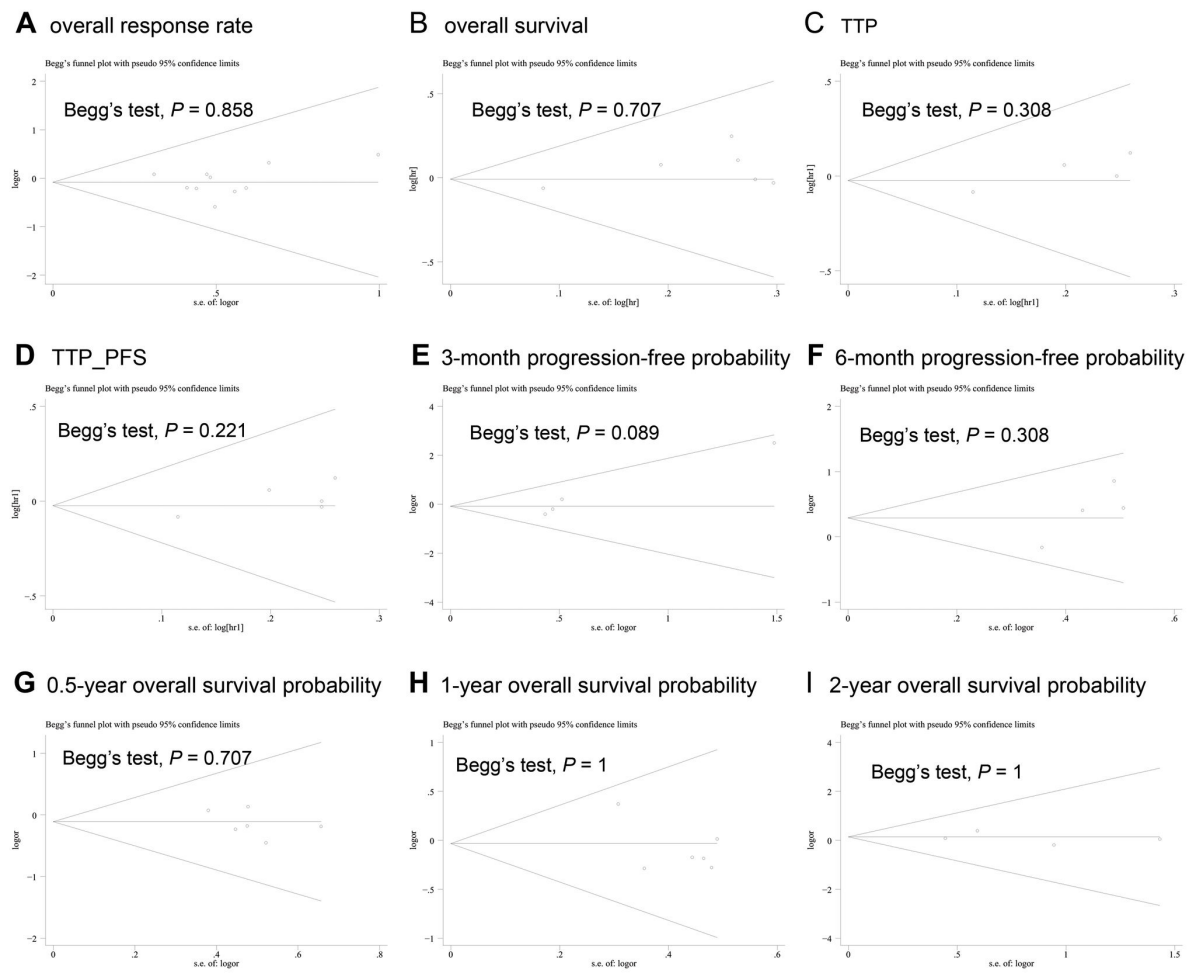
Begg’s funnel plots.

3-month progression-free probability showed borderline publication bias by Egger's test (*P* = 0.048), however, no publication bias by Begg's test (*P* = 0.089). There’s no publication bias for other results, with a symmetrical appearance on funnel plot analysis and *P* ranging from 0.221 to 1 given by Begg's test and *P* ranging from 0.102 to 0.803 by Egger's test (Figure 7). 

### Subgroup Analysis and Sensitivity Analysis

Although no significant heterogeneity was observed in all the comparisons, we probe into detail results in subgroup analyses stratified by study design (RCTs or cohort studies), patient median age (patient median age ≤ 65 or > 65), chemotherapy regimen (single drug, combined with oxaliplatin, cisplatin or docetaxel), median of chemotherapy cycles (S-1< capecitabine, S-1≥ capecitabine) and country (Japan, Korea and China). All subgroup results were quite consistent with the overall results. The subgroup analyses according to study design were showed in forest plots, while all the other subgroup analyses were summarized in Table 5. 

**Table 5:** Subgroup analysis of the meta-analysis.

Outcomes	Subgroup	No.	Effect (95%conﬁdence interval)	Estimate for overall effect	Heterogeneity
Overall response	Patient age ≤ 65	5	0.94 (0.65-1.38)	*P* = 0.77	*I* ^2^ = 0%, *P* = 0.92
rate	Patient age > 65	5	0.89 (0.55-1.43)	*P* = 0.62	*I* ^2^ = 0%, *P* = 0.82
	Single drug	3	1.04 (0.53-2.04)	*P* = 0.92	*I* ^2^ = 0%, *P* = 0.83
	Plus oxaliplatin	2	0.90 (0.49-1.66)	*P* = 0.73	*I* ^2^ = 0%, *P* = 0.73
	Plus cisplatin	4	0.92 (0.60-1.42)	*P* = 0.70	*I* ^2^ = 0%, *P* = 0.61
	Plus docetaxel	1	0.81 (0.34-1.92)	*P* = 0.63	N/A
	Cycles(S-1< Cape)	3	0.88 (0.52-1.50)	*P* = 0.65	*I* ^2^ = 0%, *P* = 0.87
	Cycles (S-1≥ Cape)	4	0.98 (0.62-1.56)	*P* = 0.93	*I* ^2^ = 0%,*P* = 0.59
	Cycles (unkown)	3	0.88 (0.50-1.54)	*P* = 0.65	*I* ^2^ = 0%,*P* = 0.93
	Japan	1	0.76 (0.26-2.27)	*P* = 0.63	N/A
	Korea	4	0.92 (0.61-1.34)	*P* = 0.63	*I* ^2^ = 0%,*P* = 0.68
	China	5	0.95 (0.60-1.60)	*P* = 0.93	*I* ^2^ = 0%,*P* = 0.94
	Overall	10	0.92 (0.69-1.24)	*P* = 0.59	*I* ^2^ = 0%,*P* = 0.98
Time to progression	Patient age ≤ 65	2	0.95 (0.78-1.16)	*P* = 0.63	*I* ^2^ = 0%, *P* = 0.54
	Patient age > 65	2	1.06 (0.75-1.51)	*P* = 0.75	*I* ^2^ = 0%, *P* = 0.73
	Single drug	1	1.00 (0.62-1.62)	*P* = 1.00	N/A
	Plus oxaliplatin	1	1.06 (0.72-1.57)	*P* = 0.77	N/A
	Plus cisplatin	2	0.95 (0.77-1.17)	*P* = 0.64	*I* ^2^ = 0%, *P* = 0.47
	Cycles (S-1< Cape)	2	1.04 (0.77-1.40)	*P* = 0.82	*I* ^2^ = 0%, *P* = 0.85
	Cycles (S-1≥ Cape)	2	0.95 (0.77-1.17)	*P* = 0.64	*I* ^2^ = 0%, *P* = 0.47
	Korea	3	1.06 (0.82-1.38)	*P* = 0.66	*I* ^2^ = 0%, *P* = 0.94
	China	1	0.92 (0.73-1.15)	*P* = 0.47	N/A
	Overall	4	0.98 (0.82-1.16)	*P* = 0.79	*I* ^2^ = 0%,*P* = 0.86
TTP_PFS	Patient age ≤ 65	3	0.96 (0.80-1.15)	*P* = 0.62	*I* ^2^ = 0%, *P* = 0.83
	Patient age > 65	2	1.06 (0.75-1.51)	*P* = 0.75	*I* ^2^ = 0%, *P* = 0.73
	Single drug	1	1.00 (0.62-1.62)	*P* = 1.00	N/A
	Plus oxaliplatin	1	1.06 (0.72-1.57)	*P* = 0.77	N/A
	Plus cisplatin	3	0.95 (0.79-1.15)	*P* = 0.63	*I* ^2^ = 0%, *P* = 0.77
	Cycles (S-1< Cape)	3	1.02 (0.79-1.32)	*P* = 0.90	*I* ^2^ = 0%, *P* = 0.96
	Cycles (S-1≥ Cape)	2	0.95 (0.77-1.17)	*P* = 0.64	N/A
	Japan	1	0.97 (0.60-1.57)	*P* = 0.90	N/A
	Korea	3	1.06 (0.82-1.38)	*P* = 0.66	*I* ^2^ = 0%, *P* = 0.94
	China	1	0.92 (0.73-1.15)	*P* = 0.47	N/A
	Overall	5	0.98 (0.83-1.15)	*P* = 0.79	*I* ^2^ = 0%, *P* = 0.94
3-month progression	Patient age ≤ 65	2	0.98 (0.50-1.93)	*P* = 0.96	*I* ^2^ = 0%, *P* = 0.56
-free probability	Patient age > 65	2	1.07 (0.51-2.25)	*P* = 0.87	*I* ^2^ =74%,*P* = 0.05
	Single drug	1	0.66 (0.28-1.56)	*P* = 0.34	N/A
	Plus oxaliplatin	1	0.82 (0.33-2.05)	*P* = 0.67	N/A
	Plus cisplatin	1	12.25(0.66-226.17)	*P* = 0.09	N/A
	Plus docetaxel	1	1.22 (0.45-3.34)	*P* = 0.70	N/A
	Cycles (S-1< Cape)	2	0.73 (0.39-1.37)	*P* = 0.33	*I* ^2^ = 0%, *P* =0 .80
	Cycles (S-1≥ Cape)	1	12.25(0.66-226.17)	*P* = 0.09	N/A
	Cycles (unkown)	1	1.22 (0.45-3.34)	*P* = 0.70	N/A
	Korea	3	0.96 (0.54-1.71)	*P* = 0.89	*I* ^2^ = 47%, *P* =0 .15
	China	1	1.22 (0.45-3.34)	*P* = 0.70	N/A
	Overall	4	1.02 (0.62-1.68)	*P* = 0.94	*I* ^2^ = 27%,*P* = 0.25
6-month progression	Patient age ≤ 65	2	1.04 (0.59-1.83)	*P* = 0.50	*I* ^2^ = 0%,*P* = 0.33
-free probability	Patient age > 65	2	1.83 (0.97-3.44)	*P* = 0.20	*I* ^2^ = 0%, *P* = 0.49
	Single drug	1	1.50 (0.64-3.49)	*P* = 0.35	N/A
	Plus oxaliplatin	1	0.85 (0.42-1.71)	*P* = 0.65	N/A
	Plus cisplatin	1	2.35 (0.90-6.14)	*P* = 0.08	N/A
	Plus docetaxel	1	1.56 (0.58-4.20)	*P* = 0.38	N/A
	Cycles (S-1< Cape)	2	1.07 (0.63-1.83)	*P* = 0.80	*I* ^2^ = 3%, *P* = 0.31
	Cycles (S-1≥ Cape)	1	2.35 (0.90-6.14)	*P* = 0.08	N/A
	Cycles (unkown)	1	1.56 (0.58-4.20)	*P* = 0.38	N/A
	Korea	3	1.74 (1.02-2.97)	*P* = 0.04	*I* ^2^ = 0%, *P* =0.76
	China	1	0.85 (0.42-1.71)	*P* = 0.65	N/A
	Overall	4	1.34 (0.88-2.04)	*P* = 0.18	*I* ^2^ = 4%, *P* = 0.37
Overall survival	Patient age ≤ 65	4	0.96 (0.84-1.11)	*P* = 0.61	*I* ^2^ = 0%, *P* = 0.93
	Patient age > 65	2	1.19 (0.83-1.72)	*P* = 0.34	*I* ^2^ = 0%, *P* = 0.70
	Single drug	1	1.11 (0.66-1.86)	*P* = 0.69	N/A
	Plus oxaliplatin	1	1.08 (0.74-1.58)	*P* = 0.69	N/A
	Plus cisplatin	4	0.97 (0.84-1.13)	*P* = 0.69	*I* ^2^ = 0%, *P* = 0.73
	Cycles (S-1< Cape)	3	1.06 (0.82-1.39)	*P* = 0.64	*I* ^2^ = 0%, *P* = 0.95
	Cycles (S-1≥ Cape)	3	0.97 (0.83-1.13)	*P* = 0.69	*I* ^2^ = 0%, *P* = 0.53
	Japan	1	0.90 (0.57-1.72)	*P* = 0.97	N/A
	Korea	4	1.11 (0.87-1.41)	*P* = 0.40	*I* ^2^ = 0%, *P* = 0.92
	China	1	0.94 (0.80-1.11)	*P* = 0.47	N/A
	Overall	6	0.99 (0.87-1.13)	*P* = 0.91	*I* ^2^ = 0%, *P* = 0.88
0.5-year survival	Patient age ≤ 65	4	0.99 (0.63-1.57)	*P* = 0.97	*I* ^2^ = 0%, *P* = 0.95
probability	Patient age > 65	2	0.72 (0.37-1.41)	*P* = 0.34	*I* ^2^ = 0%, *P* = 0.75
	Single drug	1	0.79 (0.33-1.90)	*P* = 0.60	N/A
	Plus oxaliplatin	1	1.14 (0.45-2.91)	*P* = 0.78	N/A
	Plus cisplatin	3	0.88 (0.51-1.52)	*P* = 0.66	*I* ^2^ = 0%, *P* = 0.71
	Plus docetaxel	1	0.84 (0.33-2.12)	*P* = 0.71	N/A
	Cycles (S-1< Cape)	3	0.92 (0.52-1.62)	*P* = 0.77	*I* ^2^ = 0%, *P* = 0.84
	Cycles (S-1≥ Cape)	2	0.90 (0.49-1.64)	*P* = 0.72	*I* ^2^ = 0%, *P* = 0.42
	Cycles (unkown)	1	0.84 (0.33-2.12)	*P* = 0.71	N/A
	Japan	1	0.83 (0.23-3.00)	*P* = 0.77	N/A
	Korea	4	0.92 (0.59-1.42)	*P* = 0.70	*I* ^2^ = 0%, *P* = 0.81
	China	1	0.84 (0.33-2.12)	*P* = 0.71	N/A
	Overall	6	0.90 (0.61-1.31)	*P* = 0.57	*I* ^2^ = 0%, *P* = 0.96
1-year survival	Patient age ≤ 65	4	1.03 (0.71-1.49)	*P* = 0.88	*I* ^2^ = 0%, *P* =0.53
probability	Patient age > 65	2	0.79 (0.41-1.53)	*P* = 0.49	*I* ^2^ = 0%, *P* = 0.89
	Single drug	1	0.83 (0.33-2.07)	*P* = 0.69	N/A
	Plus oxaliplatin	1	0.75 (0.37-1.51)	*P* = 0.42	N/A
	Plus cisplatin	3	1.15 (0.74-1.80)	*P* = 0.53	*I* ^2^ = 0%, *P* = 0.50
	Plus docetaxel	1	0.84 (0.35-2.01)	*P* = 0.70	N/A
	Cycles (S-1< Cape)	3	0.83 (0.52-1.34)	*P* = 0.45	*I* ^2^ = 0%, *P* = 0.89
	Cycles (S-1≥ Cape)	2	1.20 (0.72-1.98)	*P* = 0.49	*I* ^2^=23%, *P*=0.25
	Cycles (unkown)	1	0.84 (0.35-2.01)	*P* = 0.70	N/A
	Japan	1	1.01 (0.39-2.64)	*P* = 0.98	N/A
	Korea	4	0.99 (0.68-1.43)	*P* = 0.93	*I* ^2^ = 0%, *P* = 0.46
	China	1	0.84 (0.35-2.01)	*P* = 0.70	N/A
	Overall	6	0.97 (0.70-1.33)	*P* = 0.84	*I* ^2^=0%, *P*=0.74
2-year survival	Patient age ≤ 65	3	1.21 (0.61-2.37)	*P* = 0.59	*I* ^2^=0%, *P*=0.91
probability	Patient age > 65	1	0.82 (0.13-5.24)	*P* = 0.84	N/A
	Single drug	0	N/A	N/A	N/A
	Plus oxaliplatin	1	1.08 (0.45-2.60)	*P* = 0.86	N/A
	Plus cisplatin	2	1.25 (0.48-3.31)	*P* = 0.65	*I* ^2^=0%, *P*=0.60
	Plus docetaxel	1	1.05 (0.06-17.33)	*P* = 0.97	N/A
	Cycles (S-1< Cape)	2	1.22 (0.61-2.43)	*P* = 0.58	*I* ^2^=0%, *P*=0.68
	Cycles (S-1≥ Cape)	1	0.82 (0.13-5.24)	*P* = 0.84	N/A
	Cycles (unkown)	1	1.05 (0.06-17.33)	*P* = 0.97	N/A
	Japan	1	1.48 (0.46-4.72)	*P* = 0.51	N/A
	Korea	2	1.03 (0.47-2.27)	*P* = 0.94	*I* ^2^ = 0%, *P* = 0.79
	China	1	1.05 (0.06-17.33)	*P* = 0.97	N/A
	Overall	4	1.15 (0.61-2.17)	*P* = 0.66	*I* ^2^=0%, *P*=0.96

Abbreviations: **TTP_PFS**, combined time to progression and progression-free survival.

No significant heterogeneity were found in all subgroup analyses, except in patient median age > 65 subgroup analysis of 3-month progression-free probability (*I* ^2^ =74%, *P* = 0.05). We observed Seol YM recruited oldest patients among the included studies. By excluding this study, heterogeneity was reduced and the conclusion remained the same. 

### Cumulative Meta-Analysis

**Figure 8:**
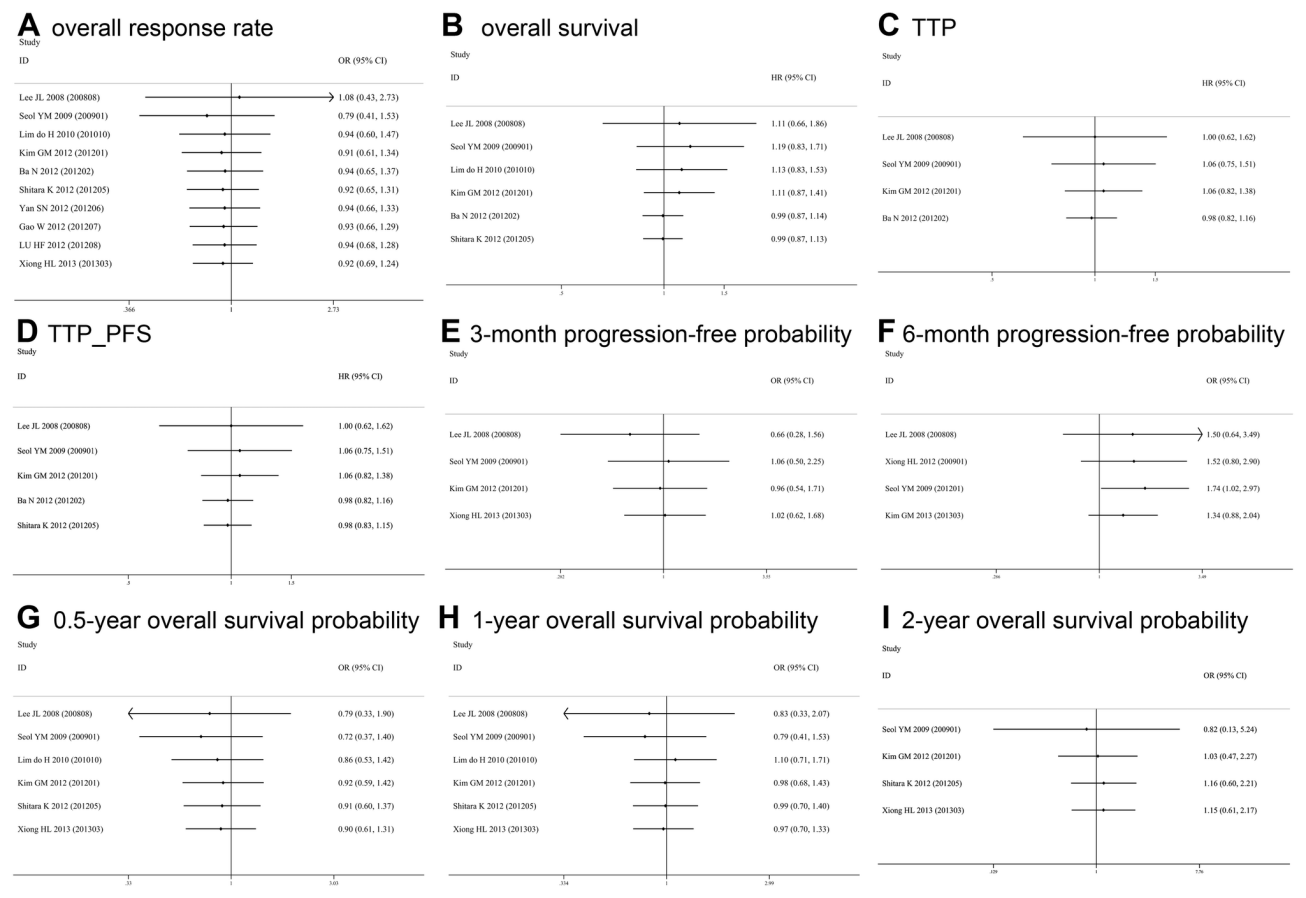
Cumulative meta-analysis to sort out the time-tendency of outcomes.

Provided time span of the available studies was considerable (from 2008-2013), a cumulative meta-analysis was encouraged to identify the time-tendency of outcomes by successively adding studies to the given result. For ORR, TTP, OS, progression-free probability and survival probability, cumulative meta-analysis consistently and stably showed equivalent effects of S-1-based chemotherapy versus capecitabine-based chemotherapy since the several initial studies were pooled, which also showed the range of 95% CI became narrower and the HR and OR were gradually closer to 1 (Figure 8).

## Discussion

As the first meta-analysis to compare S-1-based chemotherapy and capecitabine-based chemotherapy in the field of all cancers, this current meta-analysis demonstrated equivalent efficacy of the two chemotherapies as first-line treatment for AGC. A cumulative meta-analysis supported this result and suggested that the findings were robust with time. Meanwhile, equivalent tolerance was observed between two chemotherapies with regard to all the grade 3 to 4 toxicities except that hand-foot syndrome was significantly less prominent in the S-1-based chemotherapy. 

As an important measure of anti-tumor efficacy, ORR saw equivalence of S-1-based chemotherapy and capecitabine-based chemotherapy, which had a high degree of consistency with each included study. The pooled ORR (38.3%) and the ORR (20.0% to 50.0%) of S-1-based chemotherapy in the included studies were within the range of the results in the one-arm phase 1/2 clinical trials of S-1-based chemotherapy (40.0% to 54.0%) [37-42], the S-1-based arm of RCTs including FLAGS trial (29.1%) [17], SPIRITS trial (31%, 54%) [43] and the S-1-based arm of the meta-analysis comparing S-1 and 5-FU (31.3%) [19]. The dose intensity of S-1 in our included studies ranged from 168 to 560 mg/m^2^/week, between that of FLAGS trial (262.5mg/m^2^/week) and SPIRITS trial (doublet arm, 336 to 504 mg/m^2^/week) in most cases, conforming to that the ORR here is between that of FLAGS trial and SPIRITS trial (doublet arm). Similarly, the pooled ORR (39.1%) and ORR of capecitabine-based therapy (13.3% to 55.0%) were consistent with those in the one-arm phase 1/2 clinical trials of capecitabine-based chemotherapy (23.5% to 62.2%) [44-47], the capecitabine-based arm of RCT including ML17032 trial and REAL-2 trial (35% to 48%) [12,13], and the capecitabine-based arm of the meta-analysis comparing capecitabine and 5-FU (45.6%) [14,15]. The regimen of the REAL-2 trial was capecitabine (7000mg/m^2^/week) plus platinum (cisplatin 20 mg/m^2^/week or oxaliplatin 43.3 mg/m^2^/week) plus epirubicin. Although none of the 10 included studies in our meta-analysis were triplet therapy, the intensity of capecitabine-based chemotherapy in our meta-analysis was within the range of previous studies. Eight included studies had same dose intensity of doublet partner or single drug in the two arms, while 2 studies has less dose intensity of cisplatin as S-1 partner than as capecitabine partner (Lim Do H et al [20] and Shitara K et al [24]). Both studies published equivalent ORR (42% vs. 38.6%; 43.2% vs. 50%), median OS (13.3m vs. 11.2m; 13.8m vs. 13.4m) even though the dose for S-1 partner was relative less. A meta-analysis of the two studies and the sensitivity analysis after excluding the two generated same results.

The pooled HR showed comparable OS of these two therapies (HR 0.99, 95% CI 0.87-1.13) which slightly favored S-1-based therapy. As the most clinically meaningful measure of treatment effect for cancer, the impact of first-line therapy on OS may be confounded by the second-line therapy. We further accessed the chance of second-line chemotherapy and found patients with first-line S-1-based regimen received second-line chemotherapy more frequently than those with first-line capecitabine-based regimen (OR 1.82, 95% CI 1.18-2.82; *I* ^2^ = 0%). When we omitted the study with the high odds ratio of second-line chemotherapy in S-1-based regimen versus capecitabine-based regimen by Kim GM et al [22], or the study by Shitara K [24] with the high proportion of second-line chemotherapy, the comparable OS remained. Another important factor influencing OS is follow-up time. By reviewing the included studies, we found most of patients had passed away when follow-up ended and it indicated the follow-up was enough. TTP and PFS were surrogate measures for efficacy. The pooled HR for TTP and the pooled HR for TTP and PFS (TTP_PFS) [48] showed equivalent results of the two chemotherapies, with HR = 0.98 for both analyses slightly favoring S-1-based therapy. Further complementally, all equivalent 0.5-year, 1-year, 2-year overall survival probabilities, and 3-month, 6-month progression-free survival probabilities which were consistent with the case in each included study reinforced the comparable efficacy of the two therapies. 

Whether the hand-foot syndrome, thrombocytopenia, and stomatitis were more frequent for capecitabine is controversial, so is whether diarrhea occurred more for S-1 [20,23,24,33,35,36]. Our study did find a significant prominent of grade 3 to 4 hand-foot syndrome in capecitabine-based therapy versus S-1-based therapy, however, equivalent tolerance was found with regard to other grade 3 to 4 hematological and non-hematological toxicities. All 10 included studies reported the toxicities of both chemotherapies were relatively tolerable and manageable. Four studies reported decreased dose of capecitabine was largely due to hand-foot syndrome [22,23,25,35] and two studies due to hematological toxicity [22,24], however, demonstrated most patients could continue capecitabine-based therapy until progression after dose modification. The rate of 5.6% here for grade 3 or 4 hand-foot syndrome for capecitabine-based chemotherapy (based on Asian studies) was relatively lower than previous report for Westerners (11-17% in Westerners) and ethnic differences may help explain [49]. Grade 3 or 4 diarrhea was uncommon and no difference was between two chemotherapies in this meta-analysis (3.2% vs 3.6%, *P* = 0.68). Dose modification occurred unusually for S-1 and 3 studies did report due to hematological toxicity and none due to diarrhea [21,22,24]. Literature showed diarrhea was the main dose-limiting toxicity of S-1 in Westerners due to higher activity of cytochrome P-450 2A6 enzyme systems [43]. Although the dose of S-1 was reduced mainly due to diarrhea in West compared to the dose in Asia, global phase 3 FLAGS trial reported non-inferior results regarding OS between S-1 plus cisplatin and 5-FU plus cisplatin [50]. That indicated the promising value of S-1 for Westerners after careful evaluation and adjustment.

In subgroups stratified by potential confounders (regimen, age, median cycles, study design, country), equivalence of efficacy was found and was quite consistent with the overall results. This meta-analysis included 10 studies that were 3 studies of single drug, 2 studies with oxaliplatin, 4 studies with cisplatin, and 1 study with docetaxel according to regimen. Based on SPIRITS trial [43], phase 2 trials of S-1 plus oxaliplatin [39], study of capecitabine plus cisplatin and REAL-2 study [12,51], combination chemotherapy with an oral fluoropyrimidine (S-1 or capecitabine) plus a platinum (cisplatin or oxaliplatin) showed advantage over monotherapy and has been recognized as standard chemotherapy for advanced gastric cancer all over the world [52,53]. Although triplet therapy, which contains a fluoropyrimidine, a platinum, with an anthracycline or a taxane in the West, has demonstrated better or non-inferior efficacy than doublet therapy, its usage was restricted because of substantial toxicities [54,55]. That all studies included in our meta-analysis were either doublet chemotherapy or monotherapy reflected the above current situation, especially in Asia. In this background, our results delivered quite meaningful value for non-inferior S-1 plus platinum versus capecitabine plus platinum. In the subset of patients with median age > 65, 3 studies used recommended-intensity single drug (S-1, 373.3, 466.7, 560 mg/m^2^/week; capecitabine, 11666.7 mg/m^2^/week), 1 study used reduced-intensity S-1/capecitabine plus normal-intensity oxaliplatin (43.3 mg/m^2^/week). The intensity was relatively less compared to that of the median age ≤ 65 subset in which all used combination regimen. S-1/capecitabine as monotherapy or platinum plus reduced S-1/capecitabine are popular options for old patients with consideration for tolerance, especially on the basis of equivalent efficacy. Docetaxel is another promising combination partner of fluoropyrimidine, with advantage for S-1 plus docetaxel over S-1 showed in a phase 3 clinical trial [56]. A new clinical trial showed the non-inferior efficacy and toxicity of docetaxel plus S-1 than cisplatin plus S-1 [57]. Only one study about docetaxel was included in our meta-analysis, in which ORR, progression-free and survival probability were compared, but TTP and OS were not. Two meta-analyses showed docetaxel-containing palliative chemotherapy improved ORR with or without OS prolongation than non-taxane-containing for AGC [2,58]. More studies about taxane (docetaxel/pacilitaxel) combined with S-1/capecitabine are expected. 

Median number of cycles of first-line chemotherapy is one of prognostic factors for survival [59]. The median cycle ratio of S-1- vs. capecitabine-based chemotherapy ranged from 0.25 to 1.25 in the included studies. However, we noted the number of the days in a cycle differed across the included studies. Thus, when only the studies with same number of the days in one cycle for S-1- and capecitabine-based chemotherapy were considered, we still found the overall less cycles of S-1- vs. capecitabine-based therapy, which strengthened the non-inferior efficacy of the former versus the latter. In the study by Lim do H et al (S-1 > capecitabine) [20], the dose intensity of S-1 (S-1, 168 mg/m^2^/week; cisplatin, 8.6~14.3 mg/m^2^/week) was relatively less than that of the capecitabine (capecitabine, 9333.3 mg/m^2^/week; cisplatin, 8.6~33.3 mg/m^2^/week). The above two factors guarantee the comparability of two chemotherapies and resulted in equivalent efficacy and tolerance. 

Five cohort studies were included and they were of good quality with evaluation. In order to determine whether study design impact the results, we took subgroup analysis and found both RCT and cohort subsets demonstrated consistency results with overall results, except that the difference of hand-foot syndrome reach borderline significance in cohort subsets. Of all 10 included studies, half were from China, so we also took subgroup analysis according to country and draw same conclusions in Korea, Japan and China subsets as the pooled results, except the Korea subgroup for 6-momth progression-free probability favored S-1-based therapy.

Strengths of the current meta-analysis are that it was a systematic retrieval and review of the medical literature, with comprehensive exploration in subgroup analysis and cumulative analysis. All heterogeneities were insignificant. Both the fixed model and random model were used and all the results remained. However, there’re limitations in our analysis. First, as with any meta-analysis, the results were impacted by the quality of the included studies. Second, there included five RCTs and five cohort studies. The data from cohort studies might be biased and more RCTs are warranted. Third, only 1 RCT reported the study was designed by a ‘pick the winner’ format [22], however, none of the remaining 4 RCTs and 5 cohort studies reported whether it was conducted as a non-inferiority or superiority study. Efficacy was studied as the primary endpoint and toxicities as the secondary endpoint in all 10 studies. Quality of life was only referred to in 1 RCT and 1 cohort study which reported no difference as while [22,36] and none studies talked about economic costs. Only 1 RCT demonstrated the sample size (129) was of the statistical power for primary endpoint [22], and whether the sample size (30-174) had enough power to capture the endpoints for the remaining 9 studies was unclear. That was a major weakness. Therefore, on the basis of non-inferiority efficacy, more RCTs should be expected as non-inferiority trials, looking differences in terms of toxicities, quality of life or economic costs, with adequate number of patients and statistic power to capture these aspects. Forth, this meta-analysis was based on clinical studies, not the translational research. Literature showed S-1 was better in patients with high dihydropyrimidine dehydrogenase, while capecitabine was reported to be more effective in high thymidine phosphorylase gastric cancer [60,61]. A randomized study of capecitabine plus cisplatin versus S-1 plus cisplatin for AGC is ongoing in Japan, focusing on translational research [62]. This trial is expected to provide more information for choosing S-1 or capecitabine considering different translational characters. Finally, the current results are based on Asian studies, which need confirmation in the West. 

In conclusion, this meta-analysis indicated the S-1-based chemotherapy was associated with non-inferior antitumor efficacy and better safety profile, compared with capecitabine-based chemotherapy. We recommended S-1 and capecitabine can be used interchangeably for advanced gastric carcinoma, at least in Asia. Meanwhile, more high-quality randomized controlled trials and Western studies are needed to provide more information.

## Supporting Information

Checklist S1 **PRISMA Checklist.**
(DOCX)

Figure S1 **Meta-analysis of TTP_PFS for S-1-based chemotherapy compared with capecitabine-based chemotherapy.** TTP_PFS: combined time to progression and progression-free survival.
(TIF)
