# Supplementary figures and images for: S-1-Based Chemotherapy versus Capecitabine-Based Chemotherapy as First-Line Treatment for Advanced Gastric Carcinoma: A Meta-Analysis
Source: PLoS One. 2013 Dec 12;8(12):e82798. doi: 10.1371/journal.pone.0082798 (PMC3861463; doi:10.1371/journal.pone.0082798)

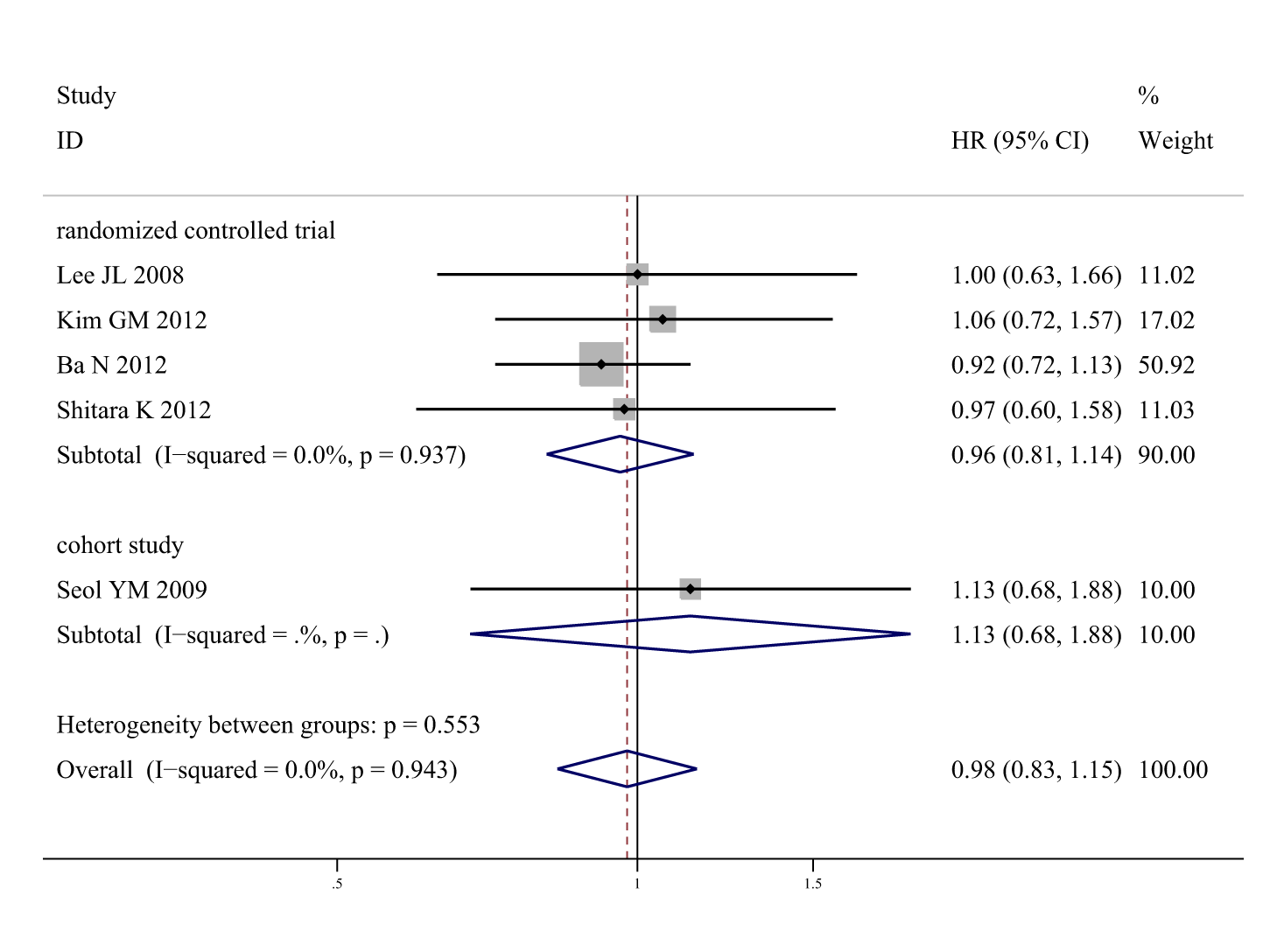

Supplement: Figure S1 — Meta-analysis of TTP_PFS for S-1-based chemotherapy compared with capecitabine-based chemotherapy. TTP_PFS: combined time to progression and progression-free survival. (TIF) [file pone.0082798.s002.tif]
